# Supplementary material for: Within-Generation Polygenic Selection Shapes Fitness-Related Traits across Environments in Juvenile Sea Bream
Source: Genes (Basel). 2020 Apr 7;11(4):398. doi: 10.3390/genes11040398 (PMC7231164; doi:10.3390/genes11040398)
Supplement: Supplementary file 1 [file genes-11-00398-s001.pdf]

Supporting Information

Supplementary Tables

Table S1. Sampling details.

Samples	Location (coordinates)	Sample Size	Sampling Date	Mean Size (mm)	Size Standard Deviation	Mean Condition
**Post larvae**	**Sète harbor** (43.392°, 3.664°)	**Total = 44**				
		25	4/10/2013	20.64	1.89	NA
		19	3/26/2013	18.25	1.39	NA
**Brackish Juveniles**	**Mauguio lagoon** (43.565°, 3.989°)	**Total = 106**				
		61	9/3/2013	164.03	19.27	1.05
		21	10/31/2013	168.86	8.54	1.16
		24	11/14/2013	163.83	11.39	1.13
**Marine Juveniles**	**Thau lagoon** (43.428°, 3.687°)	**Total = 106**				
		48	6/24/2013	47.14	10.82	1.09
		58	11/15/2013	168.90	25.79	1.07

Wild sea breams were sampled from a single population in the northern part of the Gulf of Lion, southern France (Figure 1a). We collected 3 samples from the same birth cohort (2013), consisting of pre-settled post-larvae entering coastal lagoons in early spring (N = 44), young juveniles that settled in the brackish lagoon of Mauguio (N = 106) and young juveniles that settled in the marine lagoon of Thau (N = 106).

Table S2. Pairwise genetic differentiation (F_{ST}) among the three samples based on two different polymorphism datasets (SNPs, haplotypes). Pairwise F_{ST} are larger in larval/juvenile comparisons compared the juvenile/juvenile comparison due to the smaller size of the larval sample.

Pairwise comparison	Larvae/Brackish Juv.	Larvae/Marine Juv.	Brackish Juv./Marine Juv.
Sample sizes	44/105	44/102	105/102
34679 SNPs	0.0036	0.0038	0.0027
17579 haplotypes	0.0012	0.0013	0.0008

Supplementary Methods

Test for single-generation selection

We estimated the expected distribution of allele frequency changes due to finite sample sizes in the absence of selection to generate null expectations. We considered a panmictic common gene pool of finite size (i.e. the real population) from which two samples of size $N_1$and N_2 are drawn within the same generation, either at two different times or in two different environments. For a given allele at a given bi-allelic locus, the observed allele frequency difference between the two samples ($\Delta p = |p_1 - p_2|$) can be compared with the distribution of Δp expected from random sampling effects (i.e. due to finite sample size effects). Let X be the random variable of the number of counts k for one of the two alleles in the first sample ($k \in [\![1, 2N_1 - 1]\!]$), and Z the random variable for the frequency p in the common gene pool ($p \in [0,1]$). The likelihood of observing $\{X = k\}$ allele counts in N_1 diploid individuals conditional on the allele frequency $\{Z = p\}$ in the common gene pool is given by the probability mass function of the binomial distribution:

$$\Pr(X = k|Z = p) = f(k, 2N_1, p) = \binom{2N_1}{k} p^k (1-p)^{2N_1-k}$$

From this, we evaluate the probability distribution of the unknown allele frequency $\{Z = p\}$ in the common gene pool, given that $\{X = k\}$ allele counts were observed in N_1 diploid individuals.

Using Bayes' theorem, the posterior probability distribution of Z in the common gene pool given $\{X = k\}$ observed allele counts in N_1 diploid individuals is:

$$\Pr(Z = p|X = k) = \frac{\Pr(X = k|Z = p) \times \Pr(Z = p)}{\Pr(X = k)}$$

where $\Pr(Z = p)$ is the prior probability distribution of Z in a sample of size N_1 with a genetic diversity parameter $\theta = 4N_e\mu$ (μ being the mutation rate at the examined loci and N_e the population size of the common gene pool). Assuming mutation-drift equilibrium in the common gene pool, we used equation (50) in Tajima (1989) to derive $\Pr(Z = p)$ for each value across the entire allele frequency spectrum and equation (5) to estimate θ from the data (Fig S3 & S4). The probability $\Pr(X = k)$ of observing k allele counts was given a uniform value of $1/(2N - 1)$ in $[\![1, 2N_1 - 1]\!]$.

The posterior probability distribution of Z in the common gene pool given $\{X = k\}$ observed allele counts in N_1 diploid individuals was finally used to obtain the null distribution of Δp between the two samples of size N_1 and N_2. For that, we assumed that the two samples are drawn from the same common gene pool, and used the posterior probability distribution of Z conditioned the first sample of size N_1 to predict the null distribution of allele counts in the second sample of size N_2. Finally, the distribution of allele frequency differences between the two samples was computed and compared to the observed value of Δp to estimate a P-value.

Supplementary Figures

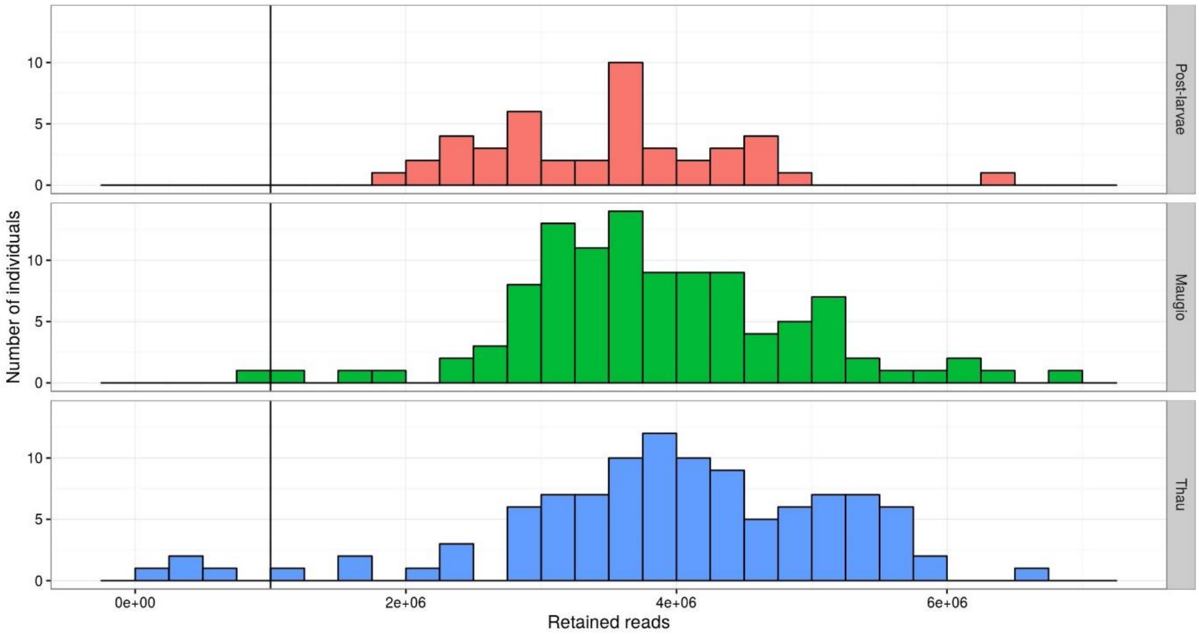

Figure S1. Distribution of per-individual read counts that were retained after quality filtering. Illumina reads were demultiplexed and quality filtered using *process_radtags* in *Stacks* and subsequently trimmed to 86 bp. This resulted in a total of 0.98×10^9 filtered reads that were used for *de novo* mapping. Five individuals were removed from the analysis due to low number of filtered reads ($<10^6$, left to vertical bar). Red: larval sample ($N = 44$), Green: brackish juveniles ($N = 106$), Blue: marine juveniles ($N = 106$).

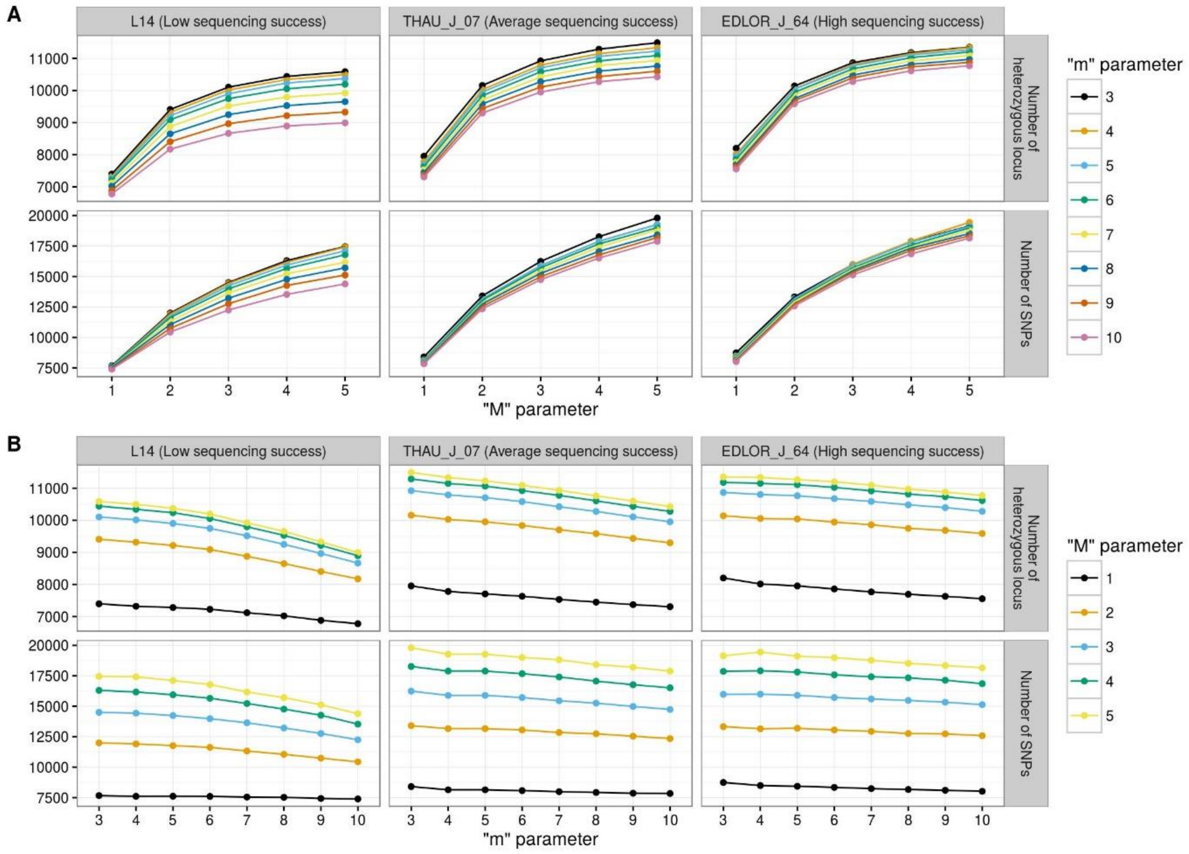

Figure S2. Parameter optimization for stacks**.** Parameters were optimized using three individuals showing low (~2.1 millions reads), average (~4,0 millions reads) and high (~6.9 millions reads) sequencing success. We used the 'bounded error rate' SNP calling model with an error rate upper bound of 1% to call individual genotypes. Putting an upper bound on the inferred error rate parameter decreases the chance of calling a homozygote when the true genotype is heterozygous, but conversely increases the potential of falsely calling a heterozygote at a homozygous locus with sequencing errors12. These parameters were also optimized using a subset of individuals to minimize the difference between the observed heterozygosity and the expected heterozygosity under Hardy-Weinberg equilibrium. A catalog of loci was then constructed with *cstacks*, allowing at most 3 mismatches (-N) between alleles within loci with the underlying hypothesis that divergence within individuals is equal to divergence between individuals, as expected under panmixia.

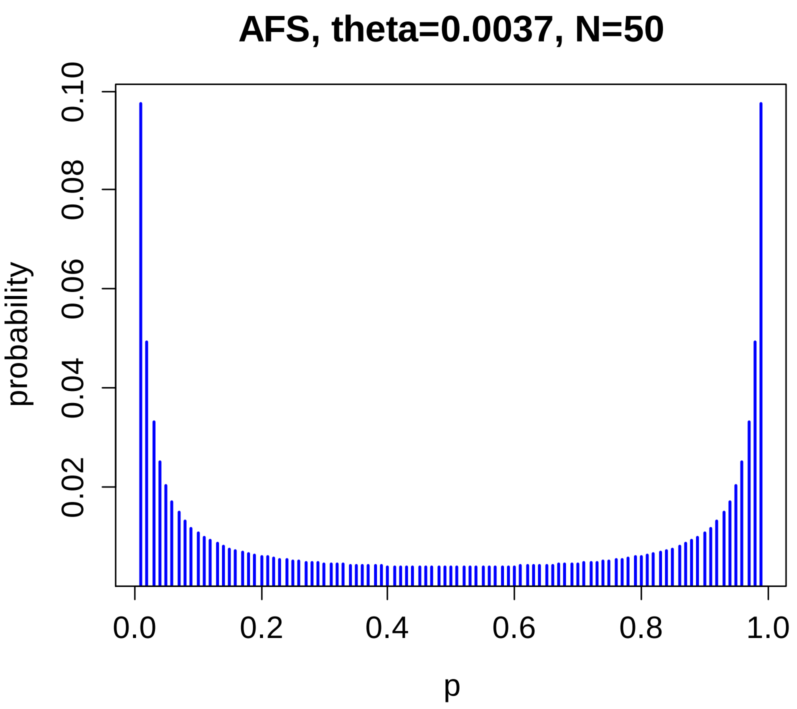

Figure S3. The prior probability distribution of Z in the common gene pool**.** Equation (50) in Tajima (1989) was used to derive the probability of each frequency value across the entire allele frequency spectrum (AFS). In this example, the sample size is $N = 50$ diploid individuals, and the genetic diversity parameter $\theta = 4N_e\mu$ has been estimated from the data using equation (5) in Tajima (1989) (The total number of individuals to estimate θ is 251, the length of each RAD locus is 86 bp, and the observed average number of SNPs per RAD locus is 2.185).

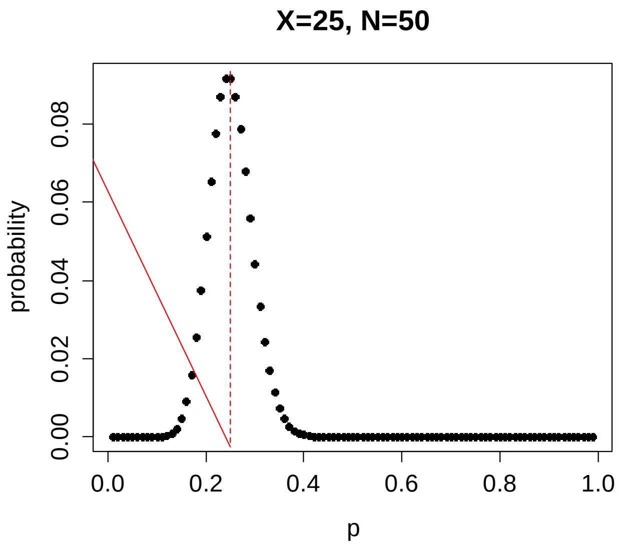

Figure S4. The posterior probability distribution of Z in the common gene pool. In this example, the number of counts for the minor allele is 25 in a sample of $N = 50$ diploid individuals. The probability distribution of the minor allele frequency in the common gene pool is centered on 0.25. The larger the sample size, the thinner the width of the distribution around the observed frequency in the sample. The genetic diversity parameter has been estimated from the data ($\theta = 0.0037$).

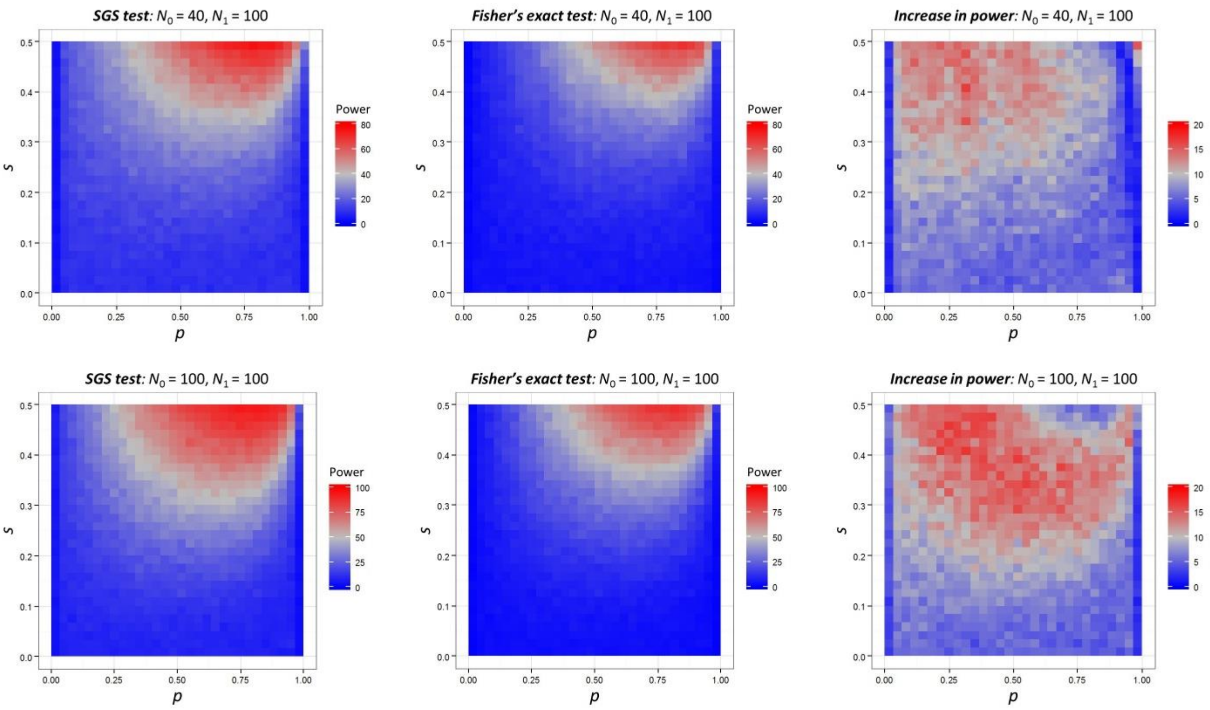

Figure S5. Assessment of the power of the Bayesian test to detect single-generation selection in comparison to Fisher's exact test. Simulations were ran to generate the expected value of Δp after one generation of selection, as a function of the initial allele frequency before selection (p) and the strength of selection (s). The population has a finite effective size ($N = 10\,000$) and genetic drift occurs after selection. The fitness of AA is $1 + s$, the fitness of Aa is 1 and the fitness of aa is $1 - s$. The population is sampled twice, once before (N_1) and after (N_2) selection and drift. A total of 100 simulations were performed for each of 101×101 combinations of starting allele frequency ($p \in [0,1]^{101}$) and selection coefficient ($s \in [0,0.5]^{101}$). For each pair of values, we determined the proportion of tests rejecting the null hypothesis of $\Delta p = 0$ at a 5% signification level, using both the Bayesian test (SGS, ***left***) and Fisher's exact test (***middle***). Simulations were performed to compare pre- and post-selection samples of size $N_1 = 40$ and $N_2 = 100$ which are close to the sampling size of larval and juvenile samples (***top***) and of size $N_1 = 100$ and $N_2 = 100$ which correspond to the juveniles sample size in brackish and lagoon habitats (***down***). The power of each test in each (p, s) pixel is indicated in color scale, as well as the increase in power provided by the SGS test in comparison to Fisher's exact test (***right***).

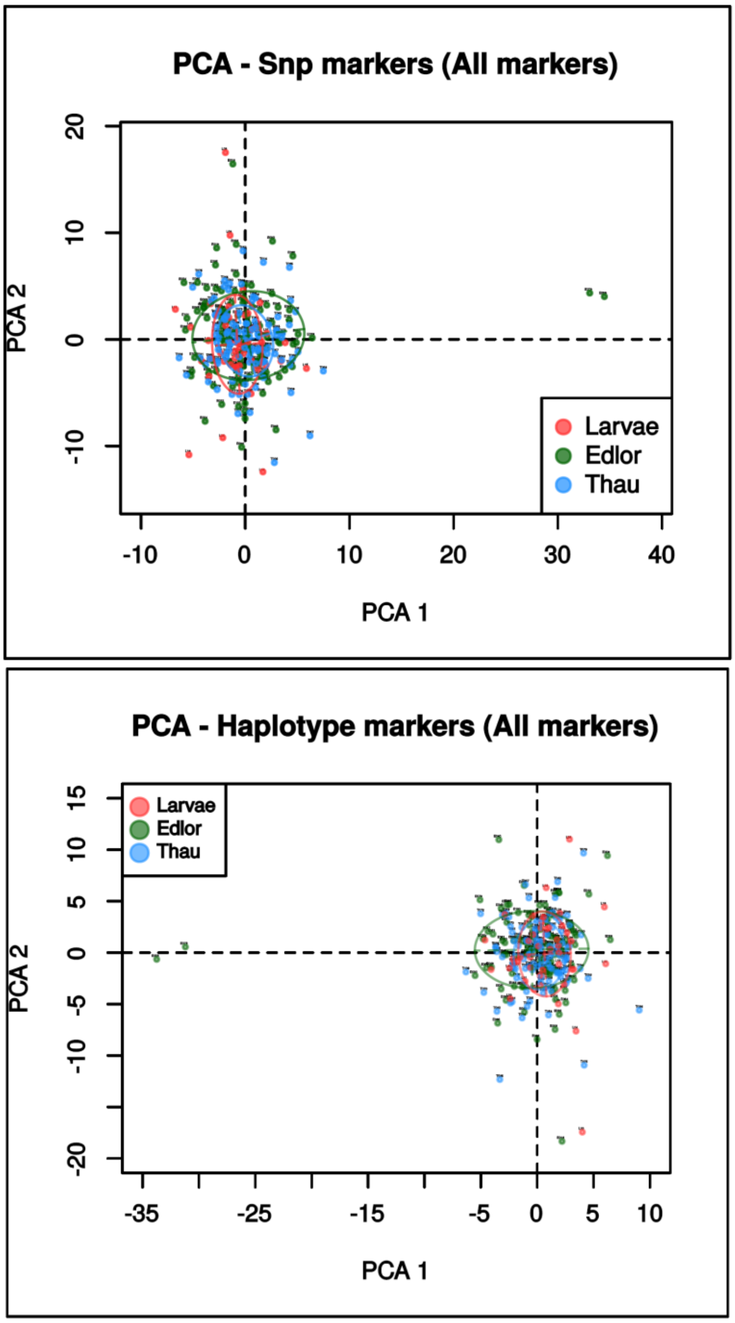

Figure S6. Principal Component Analysis performed for each of the two datasets. **Top:** using 34 679 SNPs from the de novo approach. **Middle:** using 17 579 haplotype markers from the de novo approach. Genetic diversity of larvae, marine juveniles (Thau) and brackish juveniles (Edlor) overlaps for each of the two datasets. Two juveniles from the brackish lagoon appear as outliers on the first axis of the PCA for each dataset, and probably represent closely related migrants coming from a distant population (pairwise relatedness coefficient close to 0.25).

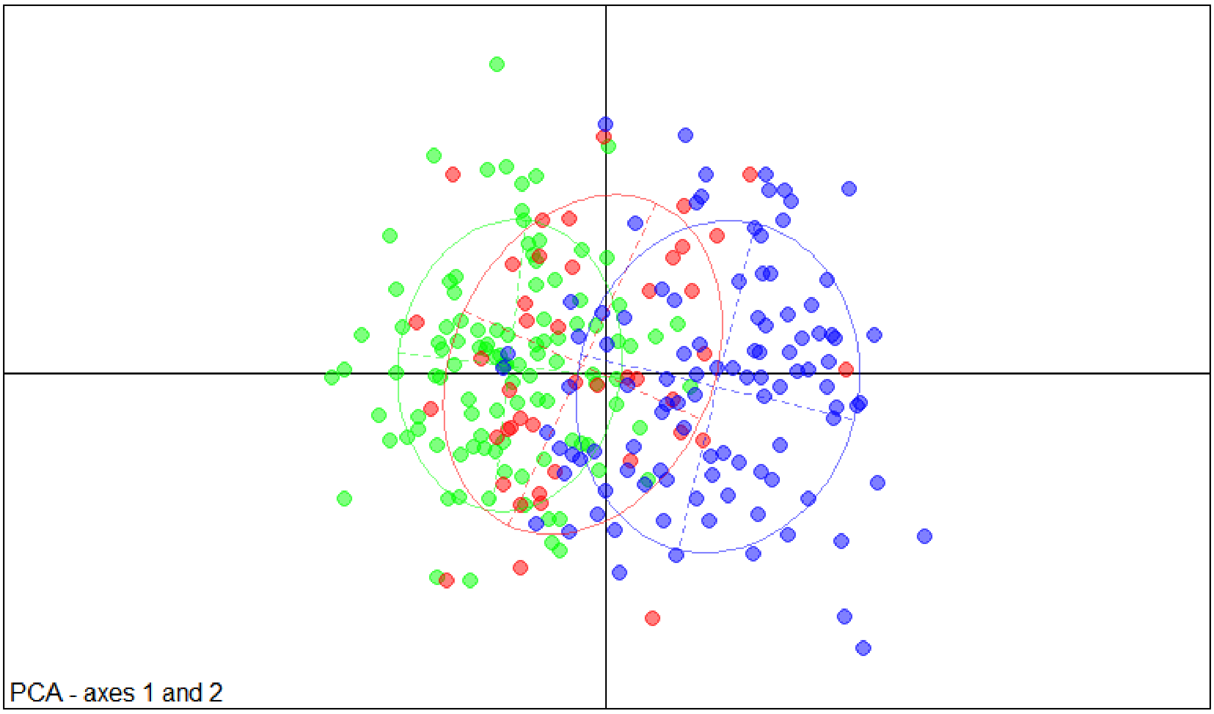

Figure S7. Principal Component Analysis performed on the outlier SNP dataset (67 SNPs from Fig. 2). Brackish (green) and marine (blue) juveniles are partly distinguished along axis 1 (horizontal). Larvae (red) lie in between, but show a diversity of genotypic combinations that mostly covers the one found in the juveniles (taking into account the fact that only 44 larvae were analyzed against 207 juveniles). Axis 1 explains 6.66% of genotype variability and axis 2 explains 4.56%.

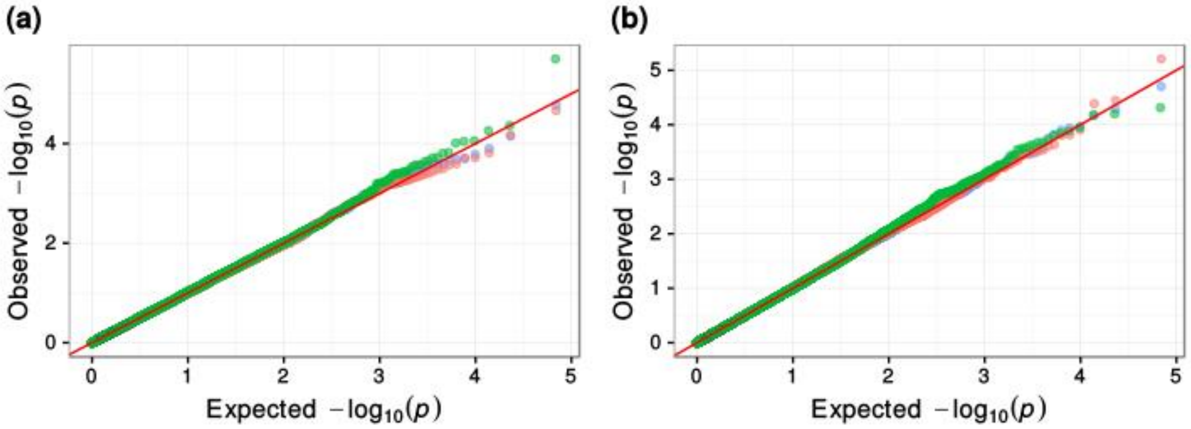

Figure S8. Genome-wide association analysis performed for standardized length (**a**) and condition (**b**). Three different models were used for each phenotype including a simple linear model (blue), a linear model including juvenile environment as a covariate (red), and a linear model including a genotype-by-environment interaction term (green).
